# Development of a Tissue-Based Extracellular Matrix Vulnerability Score (ECM-V) for Women Undergoing Primary Pelvic Organ Prolapse Surgery

**DOI:** 10.3390/biomedicines14071450

**Published:** 2026-06-26

**Authors:** Bojan Vuckovic, Milan Potic, Ivan Ignjatovic

**Affiliations:** 1 Department of Urology, Health Center Prokuplje, 18400 Prokuplje, Serbia; 2 Surgery Department, Faculty of Medicine, University of Niš, 18000 Niš, Serbia; uropota@gmail.com (M.P.); ignjatovic1964@gmail.com (I.I.); 3 Clinic of Urology, University Clinical Center Niš, 18000 Niš, Serbia

**Keywords:** pelvic organ prolapse, extracellular matrix, tissue biomarkers, matrix metalloproteinases, collagen remodeling, ECM vulnerability score, pelvic floor reconstruction, translational urogynecology, connective tissue biology

## Abstract

**Background/Objectives:** Pelvic organ prolapse (POP) is increasingly recognized as a localized extracellular matrix (ECM) remodeling disorder. Conventional clinical predictors do not fully explain interindividual variation in tissue quality or surgical durability. This study aimed to characterize the ECM failure phenotype in surgically obtained pelvic support tissue and to derive an exploratory tissue-based ECM Vulnerability (ECM-V) score. **Methods:** This single-center exploratory translational biomarker derivation study included 121 women: 60 undergoing primary reconstructive surgery for POP with or without concomitant stress urinary incontinence, and 61 benign gynecological controls. Standardized intraoperative anterior vaginal wall biopsies and preoperative plasma samples were obtained. Seven ECM biomarkers (COL1, COL3, ELN, MMP1, MMP2, MMP3, MMP9) were quantified in both compartments. Receiver operating characteristics (ROC) analysis adjusted logistic regression and stratified 10-fold cross-validation were performed. An exploratory integer-weighted ECM-V score was derived from COL3, MMP2 and MMP9 tissue values. Results: Tissue biomarkers demonstrated substantially stronger discrimination than plasma biomarkers. Surgical cases showed reduced COL1 (AUC 0.898) and ELN (AUC 0.846), elevated COL3 (AUC 0.818), MMP2 (AUC 0.958) and MMP9 (AUC 0.977) (all *p* < 0.001). The compact COL3-MMP2-MMP9 tissue model achieved a cross-validated AUC of 0.986 ± 0.035, substantially outperforming the best plasma model (AUC 0.719). The ECM-V score demonstrated derivation-level AUC of 0.995, sensitivity of 0.967 and specificity of 0.967. Tissue MMP9 and MMP2 correlated strongly with POP-Q severity and validated symptom scores (rho up to 0.806, *p* < 0.001). **Conclusions:** Women undergoing primary POP surgery demonstrate a distinct localized ECM failure phenotype. The exploratory COL3-MMP2-MMP9 framework provides a biologically coherent basis for the ECM-V score requiring prospective validation with longitudinal recurrence outcomes.

## 1. Introduction

Pelvic floor disorders, particularly pelvic organ prolapse (POP) and stress urinary incontinence (SUI), are common benign conditions with a substantial effect on daily function, sexual health, psychosocial well-being and health-care systems [1]. Incidence increases with age, vaginal childbirth, parity, obesity, menopausal status and cumulative mechanical loading [2,3]. Even though clinical exposure alone does not explain the wide variation seen in everyday practice, some women with several recognized risk factors never develop advanced pelvic floor failure, while others present with severe prolapse or recurrent dysfunction despite a less obvious clinical risk profile [4]. This discrepancy suggests that pelvic floor failure is not only an anatomical or mechanical problem but also reflects intrinsic differences in individual tissue biology.

Anatomical and biomechanical models remain central for the understanding of POP and SUI [5,6]. Vaginal delivery, multiparity, increasing age, body mass index, levator injury and an enlarged levator hiatus are linked with primary prolapse, while preoperative prolapse stage and younger age have been associated with recurrence after native tissue repair [3]. However, these predictors do not fully account for interindividual differences in disease severity, tissue quality, surgical durability or susceptibility to future failures. Patient connective tissue properties are likely to influence how mechanical forces are translated into clinically relevant pelvic support failure.

Current concepts and models view pelvic floor disorders as multifactorial conditions in which anatomical support defects, neuromuscular injury, altered biomechanics, hormonal influences, genetic predisposition and extracellular matrix (ECM) remodeling interact [7]. Pelvic support tissues are exposed throughout life to repetitive strain during pregnancy, childbirth, aging, increases in intra-abdominal pressure and daily activities. The ability to tolerate these forces and conditions depends on pelvic anatomy, but also on the quality and adaptability of the connective tissue scaffold [8].

The extracellular matrix is the main structural framework of the pelvic support tissues. Collagen type I provides tensile strength and long-term mechanical stability, while collagen type III is more closely related to tissue compliance, repair and remodeling. Elastin, by contrast, contributes to elastic recoil, allowing the tissues to recover after deformation. These components are continuously renewed through regulated matrix turnover, a process in which matrix metalloproteinases (MMPs) and their inhibitors have a main role [9]. When synthesis and degradation are imbalanced, pelvic support tissues may progressively weaken through collagen weakening, altered collagen subtype distribution, elastin loss and excessive proteolytic activity.

Histological, molecular and translational studies increasingly support the presence of altered connective tissue biology in women with POP. Reported abnormalities include reduced collagen type I expression, relative collagen type III predominance, elastin disturbance, altered collagen organization and increased matrix metalloproteinase activity in prolapsed tissues [10]. Recent systematic evidence indicates that ECM metabolism differs between women with and without prolapse, although heterogeneity between studies remains considerable [9]. Among matrix-degrading enzymes, MMP2 and MMP9 are particularly relevant because of their role in gelatin degradation, basement membrane (basal lamina) remodeling and connective tissue breakdown [11]. Together, these findings support the view that POP is not simply a mechanical displacement of pelvic organs, but a localized connective tissue remodeling disorder.

Despite this biological progress, clinically usable stratification tools remain very limited. Most previous work has described isolated biomarkers, individual tissue compartments or mechanistic pathways, without integrating these findings into a practical biological framework. In routine medical practice, decision-making within diagnostic and therapeutic frameworks still relies mainly on symptoms, POP-Q staging, compartment anatomy and conventional demographic or obstetric risk factors [6]. These measures are indeed essential, but they provide little direct information about the intrinsic vulnerability of the patient’s pelvic support tissue.

This gap is especially important when considering surgical repair. Recurrence after prolapse repair remains a major challenge in female pelvic reconstructive surgery with reported anatomical recurrence rates ranging from 20% to 35%, particularly in patients with anterior compartment defects and insufficient apical support [3,12]. Preoperative prolapse stage, levator avulsion, enlarged levator hiatal diameters and previous pelvic floor surgery are recognized as recurrence associated factors, but they still do not fully explain why apparently adequate repairs fail in some patients. One of the explanations is that recurrence partly reflects persistent intrinsic tissue vulnerability which remains present after technically successful reconstruction [7]. Characterizing pelvic support tissue at the time of the primary operation may therefore provide useful insight into the patient’s ECM phenotype.

A further distinction is needed between circulating and tissue-derived biomarkers. Plasma markers may reflect systemic ECM turnover, inflammation, vascular biology, platelet activation and metabolic variability, but they may not capture localized pelvic support pathology with adequate specificity [13]. Surgically obtained vaginal tissue, in contrast, directly samples the affected anatomical substrate involved in support failure. Tissue biomarkers are not suitable as non-invasive preoperative screening tests, but intraoperative sampling creates a valuable opportunity for biological characterization of patient’s tissue quality at the time of surgery.

The contemporary “post-mesh” era makes this question more relevant. In 2019, the United States Food and Drug Administration (FDA) ordered manufacturers to stop selling transvaginal mesh devices intended for POP repair because the available evidence did not demonstrate that benefits outweighed risks and adverse events [14]. As many reconstructive strategies now rely more heavily on native tissue support, the biological quality of the patient’s own connective tissue becomes increasingly important [12]. A tissue-based ECM profile may therefore help define a vulnerable phenotype relevant for counseling, surveillance and future recurrence-oriented research.

The present study was designed as an exploratory translational derivation study based on a clinical and biomarker dataset of women undergoing primary surgery for POP with or without concomitant SUI, compared with a benign gynecological control cohort. The study was not intended to validate a recurrence prediction model, because longitudinal recurrence outcomes were not available. Instead, we aimed to compare plasma and tissue ECM biomarkers, identify the dominant local biological signature of pelvic floor tissue failure and construct a compact tissue-based framework for patient connective tissue vulnerability characterization within initial surgery.

We hypothesized that women requiring primary surgery for POP with or without SUI would show a localized ECM failure phenotype, characterized by reduced structural matrix integrity, failure in adequate collagen remodeling, elastin depletion and increased proteolytic activity. We further hypothesized that tissue derived biomarkers would discriminate this phenotype better than plasma biomarkers and that a biologically coherent *COL3-MMP2-MMP9* axis could provide a basis for future prospective validation against true postoperative recurrence outcomes.

## 2. Materials and Methods

### 2.1. Study Design and Setting

This was a single center exploratory translational biomarker derivation study. Its purpose was to characterize the ECM phenotype associated with clinically significant pelvic floor tissue failure and to develop a biologically interpretable tissue-based framework for patient tissue vulnerability stratification.

The study was based on a prospectively collected clinical case–control biomarker dataset including women undergoing primary reconstructive pelvic floor surgery for POP with or without concomitant SUI, together with a benign gynecological surgical control cohort. The findings should be interpreted as exploratory. Because no prospective longitudinal recurrence follow-up was available, the study was not designed to establish a validated prognostic recurrence model. The aim was to characterize biological ECM vulnerability and derive a compact biologically derived ECM vulnerability score intended for future validation against postoperative recurrence outcomes.

The study was conducted in accordance with the principles of the Declaration of Helsinki. Ethical approval was obtained from the Ethics Committee of Health Center Prokuplje, Serbia (approval No. 7186, 10 October 2024), and the Ethics Committee of the Faculty of Medicine, University of Niš (approval No. 12-16442-1/2-2, 20 December 2024). Written informed consent was obtained from all participants prior to inclusion in the study.

#### 2.1.1. Study Population

A total of 121 women were included as a study population. Participants were divided into two predefined groups: surgical pelvic floor dysfunction group (*n* = 60) and benign gynecological control group (*n* = 61). The surgical group consisted of women undergoing primary reconstructive surgery for clinically significant pelvic floor dysfunction. Within this cohort, 45 women had isolated pelvic organ prolapse,15 women had combined POP and concomitant stress urinary incontinence, and no patients had isolated SUI without prolapse. Thus, the biological phenotype investigated in this study primarily reflects pelvic floor structural failure associated with prolapse biology, with SUI present only as a concomitant condition in a subset of cases. Recorded previous surgical history referred exclusively to prior non-prolapse/non-incontinence gynecological or pelvic procedures and did not include previous reconstructive surgery for POP or SUI. Therefore, all included cases represented first pelvic floor reconstructive interventions.

The control group consisted of women undergoing surgery for benign non-prolapse gynecological indications, including uterine leiomyoma, adenomyosis, abnormal uterine bleeding, and other benign gynecological conditions requiring operative treatment. Controls had no clinically significant POP, no SUI and no indication for pelvic floor reconstructive surgery. This design allowed vaginal tissue to be obtained from women without clinically evident pelvic floor structural failure, while keeping the sampling site comparable between groups.

#### 2.1.2. Inclusion and Exclusion Criteria

Surgical group inclusion criteria: Women were eligible if they were ≥18 years of age, had clinically confirmed POP with or without concomitant SUI, underwent primary surgical treatment, had standardized intraoperative tissue sampling available, had complete clinical and laboratory data with provided written informed consent.

Control group inclusion criteria: Women in control group were eligible if they were ≥18 years of age, underwent benign gynecological surgery unrelated to prolapse, had no clinically significant POP, had no stress urinary incontinence, had available standardized tissue sampling, had complete clinical and laboratory data, and provided written informed consent. 

Exclusion criteria: Participants were excluded for prior reconstructive POP/SUI surgery, pelvic malignancy, prior pelvic radiotherapy, pregnancy, active pelvic infection, systemic infection, autoimmune disease affecting connective tissue metabolism, systemic inflammatory disorders, known connective tissue disorders, chronic corticosteroid therapy, immunosuppressive treatment, inadequate tissue sampling, incomplete clinical or laboratory data.

### 2.2. Clinical and Phenotypic Assessment

Clinical and demographic variables recorded for all participants included age, body mass index (BMI), parity, number of vaginal deliveries, cesarean section history, menopausal status, symptom duration, prior pelvic surgical history. Pelvic floor anatomical assessment in surgical patients was performed using the standardized Pelvic Organ Prolapse Quantification (POP-Q) system. Symptom burden was assessed using validated pelvic floor questionnaires: Urinary Distress Inventory-6 (UDI-6) and Pelvic Floor Distress Inventory-20 (PFDI-20). POP-Q measurements and symptom scores were not used to build the derivation model, because they are closely tied to disease definition and clinical severity. They were retained for cohort description and exploratory correlation only to reduce circularity and disease definition leakage.

### 2.3. Plasma Biomarker Sampling and Examination

Peripheral venous blood was collected preoperatively before anesthesia induction and before surgical intervention. Blood was collected into EDTA-containing tubes to reduce clot-related release of matrix metalloproteinases and to preserve the plasma biomarker integrity. Samples were gently inverted, centrifuged at 3000 rpm for 10 min with plasma separated immediately, aliquoted and stored at −80 °C until examination.

The following plasma biomarkers were quantified: Collagen type I (COL1), Collagen type III (COL3), Elastin (ELN), Matrix metalloproteinase-1 (MMP1), Matrix metalloproteinase-2 (MMP2), Matrix metalloproteinase-3 (MMP3), and Matrix metalloproteinase-9 (MMP9). Biomarker concentrations were measured using commercially available ELISA kits (Wuhan Fine Biotech assay) according to manufacturer protocols. All measurements were performed in duplicate, and mean values were used for statistical processing. Plasma biomarker concentrations were expressed as ng/mL. Plasma biomarkers were considered secondary biological variables, included primarily for comparison with tissue-derived markers.

### 2.4. Tissue Sampling and Tissue Biomarker Examination

Standardized intraoperative tissue sampling was performed in all participants. A 5 × 5 mm full thickness anterior vaginal wall specimen was obtained from a standardized anatomical location approximately 30 mm lateral to the cervix on the left vaginal wall. The same anatomical site was used in cases and controls to reduce anatomical sampling variability. Tissue processing included rinsing in cold phosphate-buffered saline, removal of surface contaminants, tissue weighing, mechanical homogenization, centrifugation at 10,000 rpm for 15 min at 4 °C and supernatant collection for biomarker examination. The following tissue biomarkers were quantified: Collagen type I (COL1), Collagen type III (COL3), Elastin (ELN), Matrix metalloproteinase-1 (MMP1), Matrix metalloproteinase-2 (MMP2), Matrix metalloproteinase-3 (MMP3) and Matrix metalloproteinase-9 (MMP9). Measurements were performed using commercially available ELISA kits, (Wuhan Fine Biotech assay system) supplied through a certified local distributor. All tissue biomarker values were normalized to tissue mass and expressed as ng/mg tissue.

Both tissue and plasma biomarker measurements were performed using commercially available sandwich ELISA kits from FineTest (Wuhan Fine Biotech Co., Ltd., Wuhan, China). Manufacturer-reported analytical characteristics included detection capability, measurement interval, assay selectivity, recovery, dilution linearity, stability testing, and intra-assay/inter-assay precision as shown in Supplementary Table S1. All assays were research-use-only (RUO) assays and were not intended for diagnostic use. No CE-IVD or FDA diagnostic authorization was identified for the specific kits used. Traceability to international reference materials was not reported by the manufacturer and was not independently evaluated. Formal measurement uncertainty assessment, inter-platform comparison, and independent laboratory verification were beyond the scope of the present exploratory study.

Because the present investigation was not designed as a diagnostic accuracy study and did not evaluate a predefined index test against a clinical reference standard, formal STARD reporting was not applicable. Nevertheless, STARD principles were considered to improve transparency regarding participant selection, sample handling, assay characteristics, laboratory performance, and interpretation of discrimination metrics.

Biological domains were represented by structural matrix integrity (COL1), remodeling-associated collagen phenotype (COL3), elastic recoil (ELN), and proteolytic extracellular matrix degradation (MMP1, MMP2, MMP3, MMP9).

### 2.5. Study Endpoint and Translational Derivation Framework

Because no prospective recurrence outcome data were available, a validated prognostic recurrence endpoint could not be defined. The study therefore used surgically treated POP/POP + SUI status as a biological derivation endpoint rather than as a clinical recurrence endpoint and focused on the characterization of pelvic floor tissue failure at the extracellular matrix level.

The exploratory biological derivation endpoint was defined as the presence of clinically significant surgically treated pelvic floor dysfunction (POP ± SUI) versus absence of clinically significant pelvic floor dysfunction in controls. This endpoint was used only to identify biologically discriminatory ECM patterns and to derive a translational patient tissue vulnerability framework. It was not used to make direct recurrence predictions or postoperative failure claims.

#### Candidate Biomarker Strategy

Candidate markers were chosen to preserve biological interpretability and limit overfitting in a moderate cohort size derivation. Selection was guided by predefined mechanistic probability and observed discriminatory signal.

Primary compact tissue derivation framework included COL3 tissue, MMP2 tissue and MMP9 tissue values. These were selected because they represent maladaptive collagen remodeling with gelatinase modulated extracellular matrix degradation and dominant proteolytic tissue failure activity.

Extended exploratory tissue framework, by contrast, included COL1, COL3, ELN, MMP2 and MMP9. This model was considered exploratory because increasing predictor complexity in a moderate-sized cohort increases overfitting risk.

To avoid circular model construction, we deliberately excluded POP-Q variables, UDI-6, PFDI-20 and disease-defining symptom variables.

### 2.6. Statistical Methods

Statistical methods were implemented using IBM SPSS Statistics version 27.0 (IBM Corp., Armonk, NY, USA). Distribution normality was assessed using the Shapiro–Wilk test, supported by graphical inspection of distributions. Continuous variables were expressed as: mean ± standard deviation for normally distributed variables, median with interquartile range for non-normal distributions. Categorical variables were expressed as counts and percentages. Between-group comparisons used Student’s *t*-test, Mann–Whitney U test and chi-square test where appropriate. Associations between tissue biomarkers and clinical severity variables were explored using Spearman’s correlation coefficient. Receiver operating characteristic (ROC) analysis was used to evaluate biomarker discrimination. Reported performance metrics included apparent AUC, sensitivity, specificity and Youden index-optimized thresholds.

Because this was a derivation case–control study with biologically distinct groups, apparent performance estimates were interpreted carefully as exploratory rather than definitive measures of predictive accuracy in real-world population. Internal robustness of combined biomarker models was assessed using stratified 10-fold cross validation, with Receiver operating characteristics AUC reported as mean ± standard deviation. Because there was no external validation cohort and no longitudinal recurrence endpoint, cross-validated AUC was interpreted as an internal stability estimate rather than evidence of definitive clinical predictive accuracy.

A two-sided *p*-value < 0.05 was considered statistically significant for primary exploratory comparisons. For biomarker-panel interpretation, false discovery rate adjustment was performed using the Benjamini–Hochberg procedure, and q-values were used to identify findings that remained robust after multiple comparison controls.

#### Multivariable Modeling

Adjusted association models were constructed to evaluate whether tissue biomarker associations persisted after accounting for major clinical confounders. Adjustment variables included age, BMI and parity.

Individual tissue biomarkers were entered into adjusted logistic regression models with results reported as adjusted odds ratios, 95% confidence intervals and *p*-values. Because of the moderate cohort size and exploratory derivation design of the study, effect estimates were interpreted cautiously, particularly where wide confidence intervals suggested model instability.

### 2.7. Exploratory Tissue Vulnerability Score Derivation (ECM-V Score)

After identification of the most informative tissue markers, an exploratory tissue-based score was derived. Thresholds were determined using Receiver operating characteristics (ROC) analysis with Youden optimization. Weighted point allocation followed an intentionally simple integer structure. COL3 was assigned 1 point as a remodeling-associated collagen marker, MMP2 was assigned 2 points as a high-discrimination gelatinase marker, and MMP9 was assigned 3 points as the strongest individual proteolytic signal. The weighting reflected biological plausibility, discriminatory strength and relative statistical contribution, while preserving a format that could be tested prospectively. The resulting exploratory score used elevated COL3, elevated MMP2 and elevated MMP9 to define biologically favorable versus unfavorable extracellular matrix phenotypes. This framework is intended exclusively as an exploratory translational derivation construct and requires independent prospective validation before any clinical prognostic application.

## 3. Results

**Figure 1:**
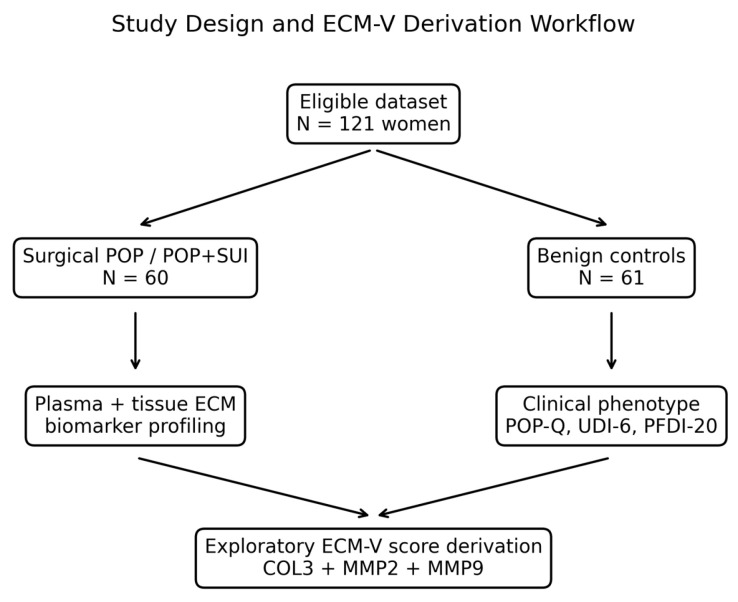
Study flowchart and exploratory translational derivation design.

The final derivation cohort included 121 women: 60 surgical cases and 61 benign gynecological controls without clinically significant POP or SUI. Among surgical cases, 45 women had isolated POP and 15 had combined POP with concomitant SUI. No patient had isolated SUI without prolapse. Recorded previous surgical history referred to non-prolapse/non-incontinence gynecological or pelvic procedures and did not include previous POP or SUI reconstructive surgery. Accordingly, all surgical cases represented first pelvic floor reconstructive interventions for clinically significant pelvic floor dysfunctions as shown in Figure 1.

### 3.1. Baseline Clinical and Phenotypic Characteristics

**Figure 2:**
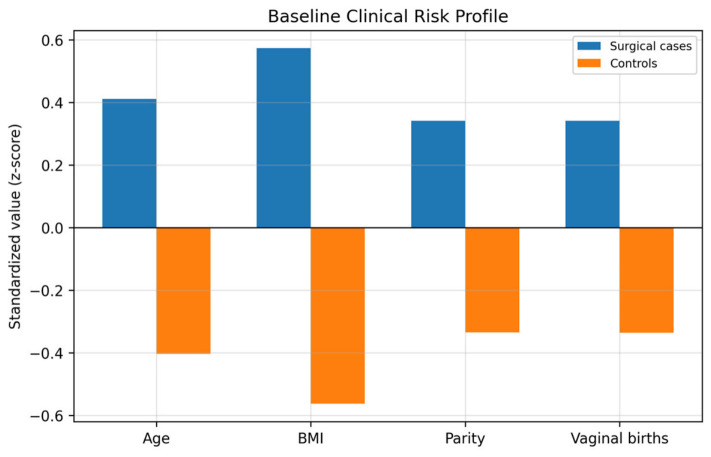
Baseline clinical risk profile of surgical cases and controls.

Surgical cases were older than controls (58.27 ± 8.24 vs. 50.95 ± 8.31 years; *p* < 0.001) and had a higher BMI (29.60 ± 3.48 vs. 25.05 ± 3.18 kg/m^2^; *p* < 0.001). Parity and vaginal delivery burden were also greater in the surgical group. Median parity was 3 (2–4) versus 2 (1–3) (*p* < 0.001), and median number of vaginal births was 2 (1–3) versus 1 (1–2) (*p* < 0.001). Cesarean section history did not differ between groups: 0 (0–1) versus 0 (0–1); *p* = 0.991. As expected, symptom burden was substantially higher in women undergoing POP/POP + SUI surgery. UDI-6 score was 31 (18–36) in cases and 0 (0–0) in controls (*p* < 0.001). PFDI-20 score was 94 (73–110) in cases and 0 (0–0) in controls (*p* < 0.001) (Table 1, Figure 2).

These variables were used for cohort description and secondary correlation assessments only and were excluded from score construction to avoid circularity.

### 3.2. Plasma Extracellular Matrix Biomarker Profile

**Figure 3:**
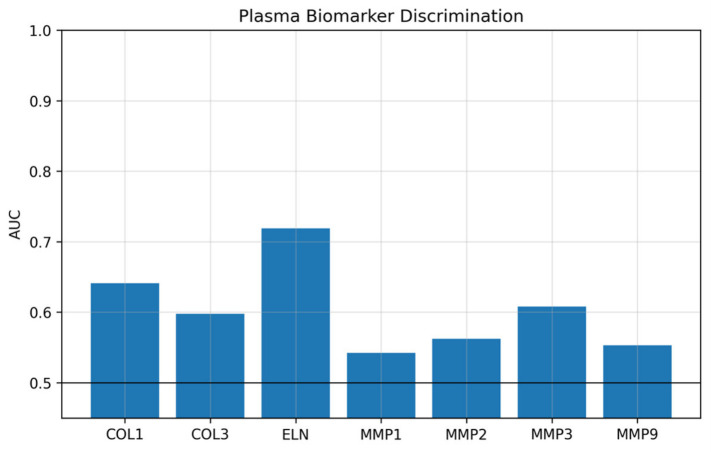
Plasma biomarker discriminatory performance (individual plasma biomarker AUCs).

Plasma biomarkers showed weaker separation between cases and controls than tissue biomarkers. Among circulating markers, plasma COL1 and plasma ELN were significantly lower in the surgical group. Plasma COL1 was 10.27 (7.69–14.20) versus 13.80 (10.20–16.30) ng/mL (*p* = 0.007), and plasma ELN was 2.49 (2.02–2.95) versus 3.20 (2.59–3.98) ng/mL (*p* < 0.001). Other circulating markers showed weaker or inconsistent signals. Plasma COL3 was 1.28 (1.04–1.68) versus 1.43 (1.36–1.72) ng/mL (*p* = 0.064), plasma MMP1 was 0.96 (0.56–1.55) versus 0.85 (0.52–1.19) ng/mL (*p* = 0.425), plasma MMP2 was 1.90 (1.13–7.51) versus 2.86 (1.64–19.65) ng/mL (*p* = 0.239), and plasma MMP9 was 2.87 (1.80–5.51) versus 3.46 (2.66–6.00) ng/mL (*p* = 0.317). Plasma MMP3 did not differ significantly between groups: 5.03 ± 2.21 versus 5.46 ± 1.64 ng/mL (*p* = 0.227) (Table 2, Figure 3).

Overall, circulating ECM changes were present but incomplete, and did not provide a sufficiently strong basis for the primary tissue-based derivation framework.

Manufacturer-reported laboratory performance characteristics of the ELISA assays used in this study are summarized in Supplementary Table S1. Across all seven biomarkers, intra-assay coefficients of variation ranged from 4.3% to 6.3%, while inter-assay coefficients of variation ranged from 4.5% to 6.3%. Recovery testing in EDTA plasma demonstrated recoveries ranging from 85% to 105%, and dilution-linearity experiments showed acceptable laboratory performance across the tested concentration ranges. All assays were commercially available research-use-only (RUO) sandwich ELISA kits supplied by a single manufacturer.

### 3.3. Tissue Extracellular Matrix Biomarker Profile

**Figure 4:**
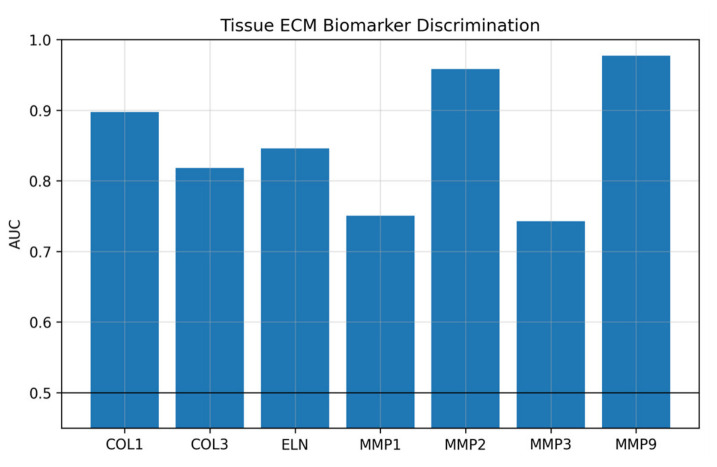
Individual tissue biomarker discriminatory performance (tissue biomarker AUCs).

Tissue-derived biomarkers showed much stronger separation between surgical cases and controls. The surgical group demonstrated a coherent ECM failure pattern, with reduced structural collagen and elastin signals, increased remodeling-associated collagen signal and increased proteolytic activity. Tissue COL1 was significantly lower in surgical cases than controls: 33.67 (29.98–38.66) versus 46.12 (43.43–50.41) ng/mg; *p* < 0.001; AUC 0.898. Tissue COL3 was significantly higher in cases: 41.11 (37.76–44.57) versus 33.23 (28.90–36.85) ng/mg; *p* < 0.001; AUC 0.818. Tissue ELN was significantly lower in cases: 21.63 (18.78–24.30) versus 27.57 (24.64–30.00) ng/mg; *p* < 0.001; AUC 0.846. Proteolytic markers were consistently elevated in the surgical group. Tissue MMP1 was higher in cases: 5.61 (4.75–6.16) versus 4.53 (3.77–5.11) ng/mg; *p* < 0.001; AUC 0.751. Tissue MMP2 demonstrated one of the strongest individual discriminatory performances: 132.44 (124.17–146.25) versus 98.72 (90.20–108.90) ng/mg; *p* < 0.001; AUC 0.958. Tissue MMP3 was also higher in cases: 17.45 ± 2.95 versus 14.83 ± 2.79 ng/mg; *p* < 0.001; AUC 0.743. Tissue MMP9 showed the strongest individual discrimination: 216.63 (197.77–237.50) versus 145.02 (133.84–160.82) ng/mg; *p* < 0.001; AUC 0.977, (Table 3, Figure 4).

Taken together, these findings indicate a localized ECM remodeling and degradation phenotype rather than an isolated abnormality of a single marker.

### 3.4. Adjusted Tissue Biomarker Associations

In order to assess whether tissue biomarker associations persisted beyond major demographic and obstetric differences, adjusted logistic regression models were constructed. Models were adjusted for age, BMI and parity. After adjustment, several tissue markers remained independently associated with surgical pelvic floor dysfunction status. Tissue COL1 showed an inverse association with case status: adjusted OR per SD 0.10; 95% CI 0.03–0.29; *p* < 0.001. Tissue COL3 remained positively associated: adjusted OR per SD 4.05; 95% CI 1.82–8.91; *p* < 0.001. Tissue ELN showed an inverse association: adjusted OR per SD 0.18; 95% CI 0.08–0.41; *p* < 0.001. Among proteolytic markers, tissue MMP2 showed a strong positive association: adjusted OR per SD 30.79; 95% CI 6.75–140.43; *p* < 0.001. Tissue MMP3 also remained independently associated: adjusted OR per SD 2.70; 95% CI 1.44–5.06; *p* = 0.002. Tissue MMP9 showed the largest adjusted effect size: adjusted OR per SD 184.29; 95% CI 13.40–2534.30; *p* < 0.001. Tissue MMP1 did not remain independently significant after adjustment: adjusted OR per SD 1.24; 95% CI 0.66–2.35; (*p* = 0.506) (Table 4).

The large effect estimates and wide confidence intervals, especially for MMP9, indicate a strong biological signal but also suggest instability compatible with near separation in this exploratory derivation cohort. These estimates should therefore be read as supportive evidence of biological relevance, not as definitive prognostic effect sizes.

### 3.5. Selection of Candidate Biomarkers for the Exploratory Tissue Failure Framework

Candidate biomarkers for the exploratory tissue-based derivation framework were selected according to biological plausibility, individual discriminatory performance and adjusted association with pelvic floor tissue failure phenotype. Based on these criteria, COL3, MMP2 and MMP9 were retained for the compact primary tissue framework. COL3 was retained as a marker of remodeling-associated collagen phenotype. MMP2 and MMP9 were retained as dominant markers of Gelatinase-mediated and proteolytic extracellular matrix degradation. COL1 and ELN were biologically important and strongly associated with case status but were reserved for the extended exploratory model because the compact 3-marker tissue framework already provided near-maximal apparent discrimination with lower model complexity. MMP1 was not retained because it did not remain independently significant after adjustment for age, BMI and parity.

#### Combined Biomarker Model Performance

**Figure 5:**
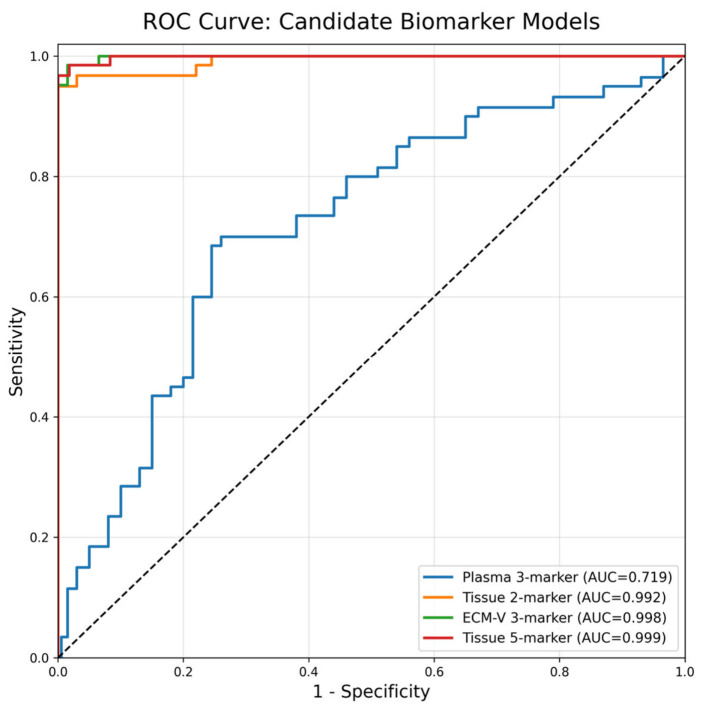
ROC comparison of plasma and tissue biomarker models.

Combined model testing confirmed the advantage of tissue-based models over plasma-based models. The 2-marker tissue model (MMP2 + MMP9) achieved an apparent AUC of 0.992, sensitivity of 0.950, specificity of 1.000 and stratified 10-fold cross-validated AUC of 0.978 ± 0.049. The compact 3-marker model (COL3 + MMP2 + MMP9) achieved an apparent AUC of 0.998, sensitivity of 0.983, specificity of 0.984 and cross-validated AUC of 0.986 ± 0.035. The extended 5-marker tissue model (COL1 + COL3 + ELN + MMP2 + MMP9) achieved an apparent AUC of 0.999, sensitivity of 0.983, specificity of 1.000 and cross-validated AUC of 0.989 ± 0.027. By contrast, the 3-marker plasma model (ELN + COL1 + MMP3) showed weaker discrimination, with apparent AUC of 0.719, sensitivity of 0.683, specificity of 0.754 and cross-validated AUC of 0.687 ± 0.219 (Table 5, Figure 5). Although the 5-marker tissue model had the numerically highest apparent AUC, its gain over the 3-marker model was minimal and required greater model complexity. The 3-marker tissue model was therefore retained as the preferred exploratory framework because it provided the best balance between parsimony, biological interpretability and discriminatory performance. Given the case–control derivation structure, these estimates should be interpreted as apparent internal discrimination and internal stability, not as validated clinical predictive accuracy. They should not be extrapolated to recurrence prediction until tested in a prospective postoperative cohort with predefined anatomical and symptomatic recurrence outcomes.

### 3.6. Exploratory Tissue Vulnerability Score (ECM-V Score)

After identifying the most informative and relevant tissue markers, we constructed a compact exploratory tissue vulnerability score (ECM-V) based on COL3, MMP2 and MMP9. Candidate thresholds were determined by Receiver operating characteristics (ROC) analysis using Youden index optimization. The resulting cut-off values were: COL3 ≥ 37.60 ng/mg, MMP2 ≥ 114.31 ng/mg and MMP9 ≥ 178.91 ng/mg. Weighted integer points were assigned according to biological relevance and individual discriminatory performance: COL3 ≥ 37.60 ng/mg = 1 point, MMP2 ≥ 114.31 ng/mg = 2 points and MMP9 ≥ 178.91 ng/mg = 3 points. This yielded a 0–6 point exploratory tissue vulnerability score (Table 6).

**Figure 6:**
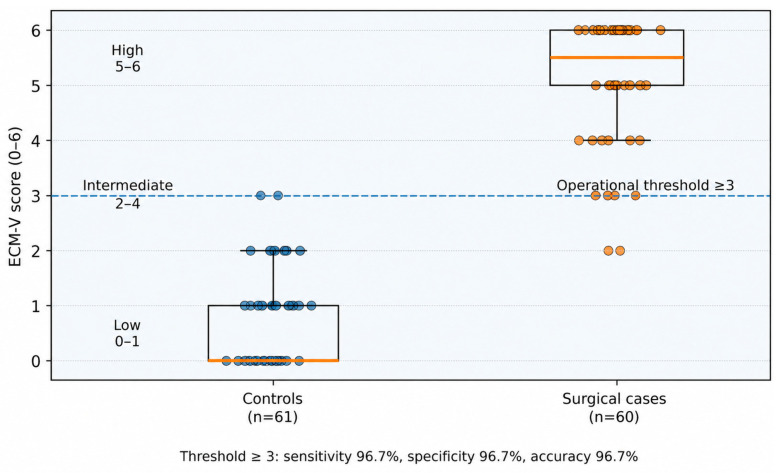
ECM-V score distribution across surgical cases and controls in the exploratory derivation cohort.

Three biological strata were defined: 0–1 points as “Low-vulnerability extracellular matrix phenotype”, 2–4 points as “Intermediate-vulnerability extracellular matrix phenotype” and 5–6 points as “High-vulnerability extracellular matrix failure phenotype” (Table 7, Figure 6).

Within the same exploratory derivation cohort, an operational threshold of ≥3 points demonstrated an AUC of 0.995, sensitivity of 0.967, specificity of 0.967 and accuracy of 0.967. In this derivation cohort, low-vulnerability scores were observed only among controls, whereas high-vulnerability scores were observed only among surgical cases. Intermediate-vulnerability scores were present in both groups, suggesting a transitional biological phenotype. This distribution should be interpreted as a derivation finding, not as independent validation of the score. Because the score was derived from a case–control dataset without longitudinal recurrence follow-up, it should be interpreted as an exploratory biological stratification tool rather than a validated recurrence-prediction score.

#### Example of ECM-V Score Application

A patient with COL3 ≥ 37.60 ng/mg, MMP2 ≥ 114.31 ng/mg and MMP9 ≥ 178.91 ng/mg receives 1 + 2 + 3 points, for a total ECM-V score of 6 and classification into the high vulnerability ECM failure phenotype. A patient below all three thresholds receives 0 points and is classified as low vulnerability, while a patient meeting only the MMP2 threshold receives 2 points and falls into the intermediate vulnerability phenotype.

### 3.7. Associations with Clinical Severity

Although POP-Q, UDI-6 and PFDI-20 were excluded from the primary derivation framework to avoid circularity, they were used as additional assessments to evaluate whether tissue biomarkers reflected clinical disease burden.

Tissue MMP9 showed strong positive correlations with POP-Q measurements: MMP9 versus POP-Q Bp, rho 0.806; *p* < 0.001; MMP9 versus POP-Q Ba, rho 0.801; *p* < 0.001; MMP9 versus POP-Q Aa, rho 0.795; *p* < 0.001; and MMP9 versus POP-Q C, rho 0.795; *p* < 0.001.

Tissue MMP2 also correlated strongly with clinical severity indicators: MMP2 versus PFDI-20, rho 0.760; *p* < 0.001; MMP2 versus POP-Q C, rho 0.752; *p* < 0.001; MMP2 versus POP-Q Ba, rho 0.719; *p* < 0.001; MMP2 versus POP-Q Aa, rho 0.719; *p* < 0.001; and MMP2 versus POP-Q Bp, rho 0.701; *p* < 0.001.

MMP9 correlated with urinary symptom burden: MMP9 versus UDI-6, rho 0.708; *p* < 0.001.

**Figure 7:**
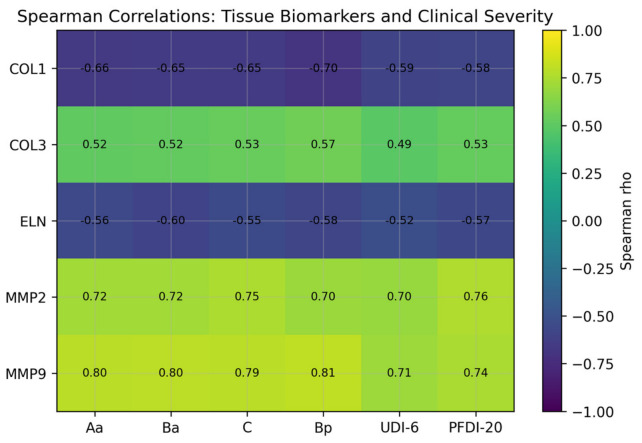
Correlation heatmap of tissue biomarkers and clinical severity parameters.

Tissue COL1 showed an inverse correlation with prolapse severity: COL1 versus POP-Q Bp, rho −0.699; *p* < 0.001 (Table 8, Figure 7).

These correlations support the clinical relevance of the tissue biomarker signal while preserving a clear separation between disease-severity variables and the exploratory tissue-failure model.

In summary, local tissue biomarkers separated surgical cases from controls much more strongly than plasma biomarkers. The dominant tissue phenotype in women undergoing primary POP/POP + SUI surgery consisted of increased COL3, MMP2 and MMP9, together with reduced COL1 and ELN. The compact COL3-MMP2-MMP9 axis showed excellent apparent discrimination and was selected as the preferred exploratory tissue vulnerability framework. Because the cohort was biologically distinct and the study did not include longitudinal recurrence endpoints, the high AUC values require cautious interpretation. The proposed score identifies a localized ECM vulnerability phenotype and requires prospective validation before any clinical recurrence prediction use can be considered.

## 4. Discussion

In this exploratory translational derivation study, we demonstrated that women undergoing primary reconstructive surgery for pelvic organ prolapse (POP), with or without concomitant stress urinary incontinence (SUI), exhibit a distinct localized extracellular matrix (ECM) failure phenotype within the affected support tissue. This phenotype was not limited to one isolated marker. It combined reduced structural collagen integrity, elastin depletion, a remodeling-dominant collagen profile and increased proteolytic activity. The quantitative separation was significantly stronger in tissue than in plasma with tissue MMP9 showing the strongest individual discrimination (AUC 0.977), followed by tissue MMP2 (AUC 0.958), tissue COL1 (AUC 0.898), tissue ELN (AUC 0.846) and tissue COL3 (AUC 0.818). Exploratory plasma models reached only modest discrimination (AUC 0.719). This pattern supports the interpretation that clinically significant pelvic floor structural failure is more strongly reflected in the affected support tissue than in systemic circulation.

Although ROC curve analysis demonstrated biologically meaningful discrimination between women with pelvic organ prolapse and controls, the present study was not designed as a diagnostic accuracy investigation. The ECM-V score was developed as an exploratory tissue-based biological framework intended to characterize extracellular matrix vulnerability rather than to establish a clinical diagnostic test. No predefined diagnostic threshold, reference interval, clinical decision limit, or intended-use diagnostic pathway was specified. Therefore, the reported discrimination metrics should be interpreted as measures of biological separation within this derivation cohort and not as evidence supporting clinical diagnostic or prognostic use.

Although the ECM-V framework demonstrated excellent discriminatory performance within the derivation cohort, these findings should be interpreted cautiously. The reported ROCs and cross-validated AUC estimates reflect apparent internal discrimination and internal stability within a biologically distinct case–control dataset rather than validated clinical predictive accuracy. Because the present study did not include longitudinal postoperative follow-up, predefined recurrence endpoints, or external validation, the ECM-V score cannot currently be interpreted as a clinical recurrence prediction tool. Instead, it should be viewed as an exploratory tissue-based biological stratification framework intended to characterize extracellular matrix vulnerability at the time of primary surgery.

Our tissue-level interpretation is consistent with broader biomechanical and epidemiological literature. Ashton-Miller and DeLancey emphasized that the female pelvic floor support depends on an integrated system of connective tissue, levator function and mechanical loading, rather than on a single anatomical element [15]. Olsen et al. showed that prolapse and incontinence surgery represent a substantial lifetime surgical burden, reporting an 11.1% lifetime risk of undergoing at least one operation for prolapse or incontinence by age 80 [16]. Wu et al. later reported that 25.0% of US women had at least one symptomatic pelvic floor disorder, including urinary incontinence, fecal incontinence or prolapse [1]. Vergeldt et al. identified parity, vaginal delivery, age and BMI as important risk factors for primary POP, while preoperative stage was the most consistently confirmed risk factor for recurrence after native tissue repair [2]. These studies frame the clinical importance of POP, but they also highlight that conventional predictors do not fully explain individual tissue failure. Our findings add a biological layer to this clinical framework by identifying measurable local ECM vulnerability.

The collagen findings are clinically significant. Collagen type I contributes tensile strength and long term mechanical durability, whereas Collagen type III is more closely associated with tissue compliance, repair and remodeling [11,17]. In the present cohort, tissue COL1 was markedly lower in surgical cases than controls (33.67 vs. 46.12 ng/mg; AUC 0.898), while tissue COL3 was higher (41.11 vs. 33.23 ng/mg; AUC 0.818). This combination suggests a shift from a mechanically durable collagen profile toward a remodeling-dominant phenotype. Kerkhof et al. in a review of connective tissue changes in POP, summarized that many studies reported increased Collagen type III, decreased Collagen type I or a reduced type I to type III ratio in prolapsed tissue [7].

Recent mechanistic data suggest that collagen remodeling in POP may reflect active biomechanical and molecular signaling pathways rather than simple passive connective tissue degeneration. This supports the theory of the biological remodeling-dominant phenotype observed in our cohort [18]. Moalli similarly reported increased Collagen III and active MMP9 expression in prolapsed vaginal tissue, interpreting this as evidence of active connective tissue remodeling rather than passive displacement alone [19]. Our data is directionally consistent with available collagen literature, while adding quantitative tissue-level discrimination within a defined clinical derivation cohort. Khadzhieva et al. further support this interpretation at the molecular expression level. In their systematic review and in bioinformatic investigation, ECM-related genes formed one of the most frequently investigated and biologically coherent groups in POP studies, with recurrent attention to Collagen, Elastin, MMPs and tissue inhibitors of metalloproteinases. Although that review emphasized heterogeneity between available studies, it reinforced the point that POP is associated with disturbed ECM organization and turnover [10]. Our study extends that concept by showing that a combined tissue phenotype with low COL1, low ELN, elevated COL3 and increased MMP2/MMP9 can be organized into a compact tissue vulnerability framework.

Elastin depletion adds another important dimension to the tissue phenotype. Elastic fibers provide recoil and recovery after deformation so that lower tissue elastin may indicate reduced capacity of pelvic support tissues to return to baseline after repetitive loading [20]. In our cohort, tissue ELN was significantly lower in cases than controls (22.63 vs. 27.57 ng/mg; AUC 0.846). Experimental and translational data support the relevance of elastic fiber biology in pelvic floor failure. Drewes et al. demonstrated POP in fibulin-5 knockout mice, linking defective elastic fiber assembly with loss of pelvic support [21]. Budatha et al. later showed that extracellular matrix proteases contribute to POP progression in mice and humans and connected fibulin-5-related mechanisms with increased MMP9 activity [22]. Zhao et al. reported decreased expression of Elastin, fibulin-5 and LOXL1 in uterosacral ligaments of women with POP, suggesting impaired elastic fiber maintenance in affected support tissues [23]. Our Elastin finding is consistent with this mechanistic axis and supports the concept of impaired elastic recoil as part of the ECM-V phenotype.

The proteolytic axis produced the strongest signal in this study. Tissue MMP2 and MMP9 showed the highest individual discriminatory performance and remained strongly associated with surgical case status after adjustment for age, BMI and parity. MMP2 reached an AUC of 0.958, and MMP9 reached an AUC of 0.977. This is biologically plausible because gelatinases participate in denatured collagen degradation, basement membrane remodeling and extracellular matrix destabilization [24]. Kerkhof et al. summarized that MMP2 and MMP9 have repeatedly been reported as increased in prolapsed tissue, while Moalli et al. linked active MMP9 expression with remodeling in prolapsed vaginal tissue [7,19]. Budatha et al. also provided mechanistic evidence that extracellular matrix proteases contribute to prolapse progression, supporting the biological relevance of proteolytic turnover in pelvic support failure [22].

The magnitude of the MMP signal, however, should be interpreted cautiously. Tissue MMP9 showed the largest adjusted effect size, but the confidence interval was wide, indicating likely instability compatible with near separation in this exploratory derivation cohort. The same caution applies to the combined tissue models. The 2-marker tissue model (MMP2 + MMP9) achieved an apparent AUC of 0.992 with cross-validated AUC 0.978 ± 0.049. The compact COL3-MMP2-MMP9 model axis achieved apparent AUC 0.998 with cross-validated AUC 0.986 ± 0.035 and the extended five-marker model achieved apparent AUC 0.999 with cross-validated AUC 0.989 ± 0.027. These figures indicate strong biological separation within the derivation cohort but should not be interpreted as validated clinical predictive performance [25,26].

The very high apparent AUC values reported in this study require explicit contextual interpretation. In a case–control derivation study with biologically distinct groups—women with clinically significant surgically treated pelvic floor dysfunction versus women without any pelvic floor pathology—the discrimination environment is inherently more favorable than would be encountered in real-world clinical practice or in a prospective recurrence prediction setting. Several methodological factors likely contributed to the observed performance levels:

First, the biological contrast between groups was maximal by design, as cases were selected for clinically significant structural failure while controls had no evidence of pelvic floor dysfunction.

Second, case–control studies are known to produce optimistic apparent discrimination estimates that do not translate directly to prospective cohort performance.

Third, the moderate cohort size increases the risk of overfitting despite cross-validation.

The stratified 10-fold cross-validated AUC values, while also high, should be interpreted as internal stability estimates rather than projections of prospective clinical performance. True clinical utility of the ECM-V framework can only be established through independent prospective validation with longitudinal recurrence endpoints in an unselected surgical population.

The contrast between plasma and tissue biomarkers is also clinically informative. Although plasma COL1 and ELN were lower in cases, the circulating signal remained incomplete and substantially weaker than the tissue signal. Plasma measurements may reflect systemic ECM turnover, inflammation, platelet activation, vascular remodeling, metabolic variability and extra-pelvic connective tissue biology [13]. Recent evidence supports the contribution of systemic inflammatory, and cytokine mediated signaling pathways in pelvic floor disorders, emphasizing the concept that circulating biomarker profiles may reflect broader systemic biological activity rather than localized pelvic support tissue pathology [27]. Tissue derived biomarkers, in contrast, directly sample the anatomical substrate involved in prolapse-related support failure. The difference between the plasma model (AUC 0.719) and the compact tissue model (AUC 0.998) therefore supports the interpretation that systemic sampling dilutes or misses the local pelvic ECM signal.

The ECM-V score was designed to preserve biological interpretability while limiting model complexity. COL3 was retained as a remodeling-associated collagen marker, MMP2 as a high-discrimination gelatinase marker and MMP9 as the dominant proteolytic tissue-failure signal. At the selected operational threshold of ≥3 points, the score demonstrated an AUC of 0.995, sensitivity of 0.967, specificity of 0.967 and accuracy of 0.967. These values should be reported transparently as derivation-level biological separation. They do not establish clinical recurrence prediction because the study did not include longitudinal recurrence outcomes [25].

The secondary correlation assessments reinforce the biological coherence of the tissue signal. Tissue MMP9 correlated strongly with anatomical prolapse severity, including POP-Q Bp (rho = 0.806), Ba (rho = 0.801), Aa (rho = 0.795) and C (rho = 0.795). Tissue MMP2 correlated with symptom burden (PFDI-20 rho = 0.760) and with several POP-Q points, while tissue COL1 showed an inverse relationship with posterior compartment severity (COL1 vs. POP-Q Bp, rho = −0.699). These correlations suggest that the observed ECM abnormalities reflect clinically meaningful disease burden rather than isolated laboratory variation.

The post-mesh reconstructive era further increases the relevance of patient tissue biology. In 2019, the U.S. Food and Drug Administration ordered manufacturers to stop selling transvaginal mesh devices intended for POP repair because the available evidence did not demonstrate that benefits outweighed risks. As reconstructive practice increasingly relies on native tissue repair, the biological quality of the patient’s own connective tissue becomes a more meaningful variable. A tissue-based ECM profile may therefore help define a vulnerable phenotype relevant for counseling, postoperative surveillance and recurrence-oriented research [12].

This study has several strengths. Plasma and tissue biomarkers were measured in the same cohort, enabling direct comparison between systemic and local biological compartments. Tissue sampling was anatomically standardized, reducing sampling heterogeneity. Major clinical confounders were addressed through adjusted assessments. In addition, the score-oriented design moves beyond purely descriptive biomarker reporting toward a structured translational framework that can be tested in future cohorts.

Formal STARD reporting was not applicable because the present investigation did not evaluate a predefined index test against a clinical reference standard and was not designed as a diagnostic accuracy study. Nevertheless, STARD principles were considered to improve transparency regarding participant selection, sample handling, assay characteristics, laboratory performance, and interpretation of discrimination metrics.

Several limitations should be acknowledged. First, this was a single-center exploratory derivation study with a moderate sample size. Second, the case–control design intentionally maximized biological contrast between groups and may therefore have inflated apparent discrimination compared with what could be expected in an unselected prospective surgical population. Third, controls were not strictly matched for age or BMI, leaving the possibility of residual confounding despite multivariable adjustment. Fourth, isolated SUI cases were not included, meaning that the observed biological profile primarily reflects prolapse-associated structural failure rather than isolated urethral dysfunction. Fifth, no longitudinal postoperative follow-up was available; therefore, anatomical recurrence, symptomatic recurrence, reoperation rates, and long-term surgical durability could not be evaluated. Finally, the ECM-V score was derived and tested within the same cohort and, despite internal cross-validation, did not undergo external validation. Consequently, the score should be interpreted as an exploratory biological stratification construct rather than a clinical decision tool or a validated recurrence-prediction model.

Also, a few laboratory limitations should be acknowledged. All biomarker measurements were performed using commercially available ELISA assays obtained from a single manufacturer, which may limit generalizability across laboratory platforms. Although manufacturer-reported validation data demonstrated acceptable sensitivity, recovery, dilution linearity, stability, and assay precision, independent laboratory validation was not performed. Traceability to international reference standards, formal measurement uncertainty assessment, and external laboratory reproducibility testing were beyond the scope of this exploratory study. Consequently, the ECM-V score should not be interpreted as a diagnostic test, prognostic model, reference range, or clinically actionable assay until independent laboratory validation, external multicenter validation, and prospective longitudinal studies have been completed.

The concept that intrinsic connective tissue biology influences surgical durability is not unique to pelvic floor reconstruction and has also been explored in other connective tissue failure conditions, such as abdominal wall hernias, where altered collagen remodeling and extracellular matrix imbalance have been associated with impaired fascial integrity and recurrence susceptibility [28]. In contrast, comparable biologically informed tissue vulnerability frameworks are not currently established in female pelvic reconstructive surgery, making the present exploratory ECM-V approach a potentially relevant translational direction.

Few studies have attempted to translate surgically obtained ECM tissue biomarkers into a structured biological vulnerability framework applicable to pelvic floor reconstruction. Most available literature has focused on molecular mechanisms, histopathology, gene expression profiling, or conventional clinical risk prediction, rather than on biologically interpretable translational stratification models [9,29]. Our study therefore provides a conceptual bridge between pelvic floor tissue biology and future individualized recurrence-oriented research. Prospective multicenter validation should test whether ECM-V, alone or combined with clinical predictors, pelvic floor imaging, levator injury assessment and additional molecular markers, improves individualized risk stratification.

Overall, these findings support the view that clinically significant pelvic floor failure is a localized ECM remodeling disorder involving structural collagen depletion, maladaptive collagen remodeling, elastin loss and dominant proteolytic degradation. The COL3-MMP2-MMP9 axis framework should therefore be considered an exploratory, biologically grounded platform for future validation, not a finalized prognostic model.

## 5. Conclusions

This exploratory translational biomarker derivation study indicates that women undergoing primary surgery for POP, with or without concomitant SUI, have a distinct localized ECM failure phenotype. The phenotype includes reduced structural collagen integrity, elastin depletion, maladaptive collagen remodeling and dominant proteolytic ECM degradation. Tissue-derived markers discriminated against this pattern more strongly than circulating biomarkers, supporting the concept that clinically significant pelvic floor structural failure is best captured at the level of the affected support tissue.

Among the evaluated markers, tissue COL3, MMP2 and MMP9 formed the most coherent compact framework. Together, they represent complementary domains of ECM dysfunction as a remodeling-associated collagen shift and proteolytic tissue degradation. These findings support the biological plausibility of patient’s connective tissue vulnerability as a contributor to pelvic floor structural failure beyond conventional demographic and anatomical risk factors.

The study does not establish a validated recurrence prediction model. Instead, it provides an exploratory derivation framework suggesting that biological characterization of surgically obtained pelvic support tissue at the primary operation may offer a useful approach to host tissue stratification. This work provides an early translational framework linking extracellular matrix biology with clinically meaningful pelvic floor failure, offering a foundation for future precision approaches in female pelvic reconstructive surgery. In a reconstructive landscape increasingly focused on native tissue durability, biologically informed tissue characterization may contribute to future surveillance strategies, counseling and recurrence-oriented research. Prospective multicenter studies with longitudinal recurrence outcomes are required before clinical utility can be established. These findings position localized ECM biology as more than a mechanistic feature of pelvic floor dysfunction; they identify it as a promising translational domain for future individualized female pelvic reconstructive medicine.

Rather than representing a definitive predictive tool, the ECM-V framework should be viewed as an initial step toward biologically informed personalization in female pelvic reconstructive surgery, where intrinsic tissue quality may eventually complement traditional anatomical and clinical risk assessment.

Not all pelvic floors fail for the same biological reasons. If prospectively validated, recognition of this intrinsic connective tissue vulnerability may help move female pelvic reconstructive surgery toward truly individualized care.

## Figures and Tables

**Table 1:** Baseline clinical and phenotypic characteristics of the derivation cohort.

Variable	Cases	Controls	Test	Statistic	*p*
Age (years)	58.27 ± 8.24	50.95 ± 8.31	Student’s *t*-test	t = 4.863	<0.001
BMI (kg/m^2^)	29.60 ± 3.48	25.05 ± 3.18	Student’s *t*-test	t = 7.527	<0.001
Parity	3 (2–4)	2 (1–3)	Mann–Whitney U	U = 1167.50	<0.001
Vaginal births	2 (1–3)	1 (1–2)	Mann–Whitney U	U = 1181.50	<0.001
Cesarean section	0 (0–1)	0 (0–1)	Mann–Whitney U	U = 1827.50	0.988
UDI-6 score	31 (18–36)	0 (0–0)	Mann–Whitney U	U = 0.00	<0.001
PFDI-20 score	94 (73–110)	0 (0–0)	Mann–Whitney U	U = 0.00	<0.001

Note: Values are presented as mean ± standard deviation (SD) for variables evaluated using Student’s *t*-test, and as median (interquartile range) for variables evaluated using the Mann–Whitney U test.

**Table 2:** Comparison of plasma biomarkers between groups.

Variable	Cases	Controls	Test	Statistic	*p*
COL1 plasma (ng/mL)	10.27 (6.98)	13.81 (6.47)	Mann–Whitney U	U = 2347.00	0.007
COL3 plasma (ng/mL)	1.28 (0.69)	1.43 (0.36)	Mann–Whitney U	U = 2187.50	0.064
ELN plasma (ng/mL)	2.49 (0.95)	3.20 (1.42)	Mann–Whitney U	U = 2631.50	<0.001
MMP1 plasma (ng/mL)	0.96 (1.13)	0.85 (0.69)	Mann–Whitney U	U = 1675.50	0.423
MMP2 plasma (ng/mL)	1.90 (6.66)	2.86 (20.43)	Mann–Whitney U	U = 2057.50	0.238
MMP3 plasma (ng/mL)	5.03 ± 2.21	5.46 ± 1.64	Student’s *t*-test	t = −1.214	0.227
MMP9 plasma (ng/mL)	2.87 (3.96)	3.46 (3.42)	Mann–Whitney U	U = 2023.50	0.316

Note: Values are presented as mean ± standard deviation (SD) for variables evaluated using Student’s *t*-test, and as median (interquartile range) for variables evaluated using the Mann–Whitney U test.

**Table 3:** Comparison of tissue biomarkers between groups and their diagnostic performance.

Variable	Cases	Controls	Test	Statistic	*p*	AUC	AUC *p*
COL1 tissue (ng/mg)	33.67 (9.05)	46.12 (7.36)	Mann–Whitney U	U = 3285.00	<0.001	0.898	<0.001
COL3 tissue (ng/mg)	41.11 ± 6.00	33.23 ± 5.78	Student’s *t*-test	t = 7.367	<0.001	0.818	<0.001
ELN tissue (ng/mg)	21.63 ± 4.24	27.57 ± 3.92	Student’s *t*-test	t = −8.014	<0.001	0.846	<0.001
MMP1 tissue (ng/mg)	5.61 ± 1.16	4.53 ± 1.17	Student’s *t*-test	t = 4.822	<0.001	0.751	<0.001
MMP2 tissue (ng/mg)	132.44 (22.96)	98.72 (19.63)	Mann–Whitney U	U = 153.00	<0.001	0.958	<0.001
MMP3 tissue (ng/mg)	17.45 ± 2.95	14.83 ± 2.79	Student’s *t*-test	t = 5.018	<0.001	0.743	<0.001
MMP9 tissue (ng/mg)	216.63 (40.91)	145.02 (27.80)	Mann–Whitney U	U = 84.50	<0.001	0.977	<0.001

Note: Values are presented as mean ± standard deviation (SD) for variables evaluated using Student’s *t*-test, and as median (interquartile range) for variables evaluated using the Mann–Whitney U test.

**Table 4:** Adjusted logistic models for tissue biomarkers.

Marker	Adjusted OR per 1 SD (95% CI)	Adjusted *p*	Univariate AUC	Adjusted Model AUC
COL1 tissue (ng/mg)	0.10 (0.03–0.29)	<0.001	0.898	0.963
COL3 tissue (ng/mg)	4.05 (1.82–8.99)	<0.001	0.818	0.930
ELN tissue (ng/mg)	0.18 (0.08–0.41)	<0.001	0.846	0.946
MMP1 tissue (ng/mg)	1.24 (0.65–2.36)	0.506	0.751	0.905
MMP2 tissue (ng/mg)	30.79 (6.75–140.43)	<0.001	0.958	0.980
MMP3 tissue (ng/mg)	2.71 (1.44–5.09)	0.002	0.743	0.923
MMP9 tissue (ng/mg)	184.29 (13.40–2534.30)	<0.001	0.977	0.988

**Table 5:** Combined model performance and selection of the final derivation framework.

Model	AUC	Sensitivity	Specificity	10-Fold CV AUC
Plasma 3-marker (ELN + COL1 + MMP3)	0.719	0.683	0.754	0.687 ± 0.219
Tissue 2-marker (MMP2 + MMP9)	0.992	0.950	1.000	0.978 ± 0.049
Tissue 3-marker (COL3 + MMP2 + MMP9)	0.998	0.983	0.984	0.986 ± 0.035
Tissue 5 marker (COL1 + COL3 + ELN + MMP2 + MMP9)	0.999	0.983	1.000	0.989 ± 0.027

**Table 6:** Exploratory ECM-vulnerability score structure.

Marker	Cut-Off	Direction	Biological Meaning	Score Contribution
COL3 tissue	≥37.60 ng/mg	High adverse	Remodeling-associated collagen phenotype	1 point
MMP2 tissue	≥114.31 ng/mg	High adverse	Gelatinase-mediated ECM degradation	2 points
MMP9 tissue	≥178.91 ng/mg	High adverse	Dominant proteolytic tissue-failure signal	3 points

**Table 7:** ECM-V score (Tissue-failure phenotype score).

Total Score	Biological Stratum	Interpretation
0–1	Low-vulnerability ECM phenotype	Biologically favorable tissue profile
2–4	Intermediate-vulnerability ECM phenotype	Transitional tissue-remodeling profile
5–6	High-vulnerability ECM failure phenotype	Biologically unfavorable tissue profile

**Table 8:** Secondary Spearman correlations between tissue biomarkers and clinical severity variables.

Biomarker	Clinical Variable	Spearman Rho	*p*-Value
MMP9 tissue	POP-Q Bp	0.806	<0.001
MMP9 tissue	POP-Q Ba	0.801	<0.001
MMP9 tissue	POP-Q Aa	0.795	<0.001
MMP9 tissue	POP-Q C	0.795	<0.001
MMP2 tissue	PFDI-20	0.760	<0.001
MMP2 tissue	POP-Q C	0.752	<0.001
MMP2 tissue	POP-Q Ba	0.719	<0.001
MMP2 tissue	POP-Q Aa	0.719	<0.001
MMP2 tissue	POP-Q Bp	0.701	<0.001
MMP9 tissue	UDI-6	0.708	<0.001
COL1 tissue	POP-Q Bp	−0.699	<0.001

## Data Availability

The anonymized raw dataset, SPSS data files, and supporting statistical outputs used in this study are publicly available in the Zenodo repository at https://doi.org/10.5281/zenodo.20369870.

## Supplementary Materials

The following supporting information can be downloaded at: https://www.mdpi.com/article/10.3390/biomedicines14071450/s1, Table S1: Manufacturer-reported examination characteristics of ELISA assays used in the study.

## Abbreviations

The following abbreviations are used in this manuscript:

AUC	Area Under the Curve
Aa	Anterior vaginal wall point Aa (POP-Q)
Ba	Anterior vaginal wall point Ba (POP-Q)
BMI	Body Mass Index
Bp	Posterior vaginal wall point Bp (POP-Q)
CI	Confidence Interval
COL1	Collagen Type I
COL3	Collagen Type III
CV	Cross-Validation
ECM	Extracellular Matrix
ECM-V	Extracellular Matrix Vulnerability Score
EDTA	Ethylenediaminetetraacetic Acid
ELISA	Enzyme-Linked Immunosorbent Assay
ELN	Elastin
FDA	United States Food and Drug Administration
MMP	Matrix Metalloproteinase
MMP1	Matrix Metalloproteinase-1
MMP2	Matrix Metalloproteinase-2
MMP3	Matrix Metalloproteinase-3
MMP9	Matrix Metalloproteinase-9
OR	Odds Ratio
PBS	Phosphate-Buffered Saline
PFDI-20	Pelvic Floor Distress Inventory-20
POP	Pelvic Organ Prolapse
POP-Q	Pelvic Organ Prolapse Quantification System
ROC	Receiver Operating Characteristic
rpm	Revolutions Per Minute
SD	Standard Deviation
SPSS	Statistical Package for the Social Sciences
SUI	Stress Urinary Incontinence
UDI-6	Urinary Distress Inventory-6
