# Supplementary material for: Development of a Tissue-Based Extracellular Matrix Vulnerability Score (ECM-V) for Women Undergoing Primary Pelvic Organ Prolapse Surgery
Source: Biomedicines. 2026 Jun 26;14(7):1450. doi: 10.3390/biomedicines14071450 (PMC13405401; doi:10.3390/biomedicines14071450)
Supplement: Supplementary file 1 [file biomedicines-14-01450-s001.zip › biomedicines-4367235-supplementary.pdf]

Supplementary Table S1. Manufacturer-reported examination characteristics of ELISA assays used in the study

Biomarker	Catalog No.	Measurement Range	Sensitivity	Intra-assay CV (%)	Inter-assay CV (%)	EDTA Plasma Recovery (%)	EDTA Plasma Linearity (%)	Stability	Regulatory Status
**COL1A1**	EH0958	0.313–20 ng/mL	0.188 ng/mL	4.69–5.72	4.88–5.54	85–99 (avg 93)	85–93 (1:2), 85–98 (1:4), 80–95 (1:8)	80%; 95–100%; 85–98%	RUO
**COL3A1**	EH0746	31.25–2000 pg/mL	18.75 pg/mL	4.90–5.91	5.94–6.23	91–105 (avg 97)	91–99 (1:2), 84–95 (1:4), 85–98 (1:8)	80%; 95–100%; 85–98%	RUO
**ELN**	EH1505	0.469–30 ng/mL	0.281 ng/mL	4.33–5.12	4.80–5.45	87–105 (avg 96)	87–100 (1:2), 85–99 (1:4), 86–99 (1:8)	80%; 95–100%; 85–98%	RUO
**MMP-1**	EH0232	78.125–5000 pg/mL	46.875 pg/mL	4.92–5.13	4.81–5.09	87–99 (avg 93)	83–99 (1:2), 83–87 (1:4), 83–96 (1:8)	80%; 95–100%; 85–98%	RUO
**MMP-2**	EH0017	0.5–32 ng/mL	0.191 ng/mL	4.51–5.62	4.55–5.33	90–105 (avg 97)	83–97 (1:2), 88–98 (1:4), 83–100 (1:8)	80%; 95–100%; 85–98%	RUO
**MMP-3**	EH0235	0.156–10 ng/mL	0.094 ng/mL	4.95–6.32	5.54–6.20	92–100 (avg 97)	98–104 (1:2), 85–103 (1:4), 86–103 (1:8)	80%; 95–100%; 85–98%	RUO
**MMP-9**	EH0238	0.313–20 ng/mL	0.188 ng/mL	4.69–5.89	4.88–5.23	87–105 (avg 98)	83–99 (1:2), 84–95 (1:4), 83–100 (1:8)	80%; 95–100%; 85–98%	RUO

All assays were commercially available sandwich ELISA kits supplied by FineTest (Wuhan Fine Biotech Co., Ltd., Wuhan, China). The manufacturer reports assay selectivity as "no obvious cross-reaction with other analogues" for all included biomarkers. Precision testing was performed using 20 replicate measurements on the same plate (intra-assay precision) and 20 replicate measurements on three different

plates (inter-assay precision). All assays were designated for research use only (RUO) and were not intended for diagnostic use. No CE-IVD or FDA diagnostic authorization was identified for the specific assays used. Traceability to international reference materials, formal measurement uncertainty assessment, and independent inter-platform analytical validation were not reported by the manufacturer and were not independently evaluated in the present study. Laboratory validation data were manufacturer-reported and were not independently verified by the investigators.
